# Engineering Marrow-Mimetic Hydrogel Platforms Enhance Erythropoiesis: A Mechanobiology-Driven Approach for Transfusion Red Blood Cell Production

**DOI:** 10.3390/gels11080594

**Published:** 2025-07-31

**Authors:** Qinqin Yang, Runjin Liu, Xiang Wang

**Affiliations:** Key Laboratory of Biorheological Science and Technology, Ministry of Education, College of Bioengineering, Chongqing University, Chongqing 400044, China; 13678421641@163.com (Q.Y.); lrj990315@163.com (R.L.)

**Keywords:** bone marrow hematopoietic stem cells, erythroid differentiation, gelatin methacryloyl -poly (ethylene glycol) diacrylate, matrix stiffness

## Abstract

Red blood cell (RBC) production from bone marrow hematopoietic stem cells (BMHSCs) in vitro overlooks the mechanical signals of the bone marrow niche and overly relies on growth factors. Considering that the fate of hematopoietic stem cells (HSCs) is determined by the natural bone marrow microenvironment, differences in mechanical microenvironments provide a reference for the regulation of HSC differentiation. This study seek to reveal the role of mechanobiology cues in erythropoiesis and provide a new perspective for the design of in vitro erythropoiesis platforms. The hydrogel platforms we designed simulate the stiffness gradient of the bone marrow niche to culture HSCs and induce their differentiation into the erythroid system. Cells on the low-stiffness scaffold have higher potential for erythrocyte differentiation and faster differentiation efficiency and promote erythrocyte differentiation after erythropoietin (EPO) restriction. In vivo transplantation experiments demonstrated that these cells have the ability for continuous proliferation and differentiation into mature erythrocytes. By combining mechanical cues with in vitro erythrocyte production, this method is expected to provide insights for in vitro hematopoietic design and offer a scalable cell manufacturing platform for transfusion medicine.

## 1. Introduction

In current clinical practice, red blood cell (RBC) transfusions are indispensable for treating anemia, trauma, and chronic diseases. However, donor-dependent RBC supplies face critical shortages due to limited donations, logistical constraints, and incompatibility risks [1]. To overcome this limitation, scalable in vitro RBC production from hematopoietic stem cells (HSCs) represents a promising solution [2]. However, existing in vitro methods fail to achieve the amplification efficiency and differentiation kinetics required for clinical translation [3]. Therefore, it is crucial to explore new amplification principles. Recent studies have shown that HSCs are highly sensitive to their mechanical microenvironment [4,5], and these mechanical signals directly affect the self-renewal, differentiation, survival, and expression of molecules related to HSCs [6,7]. In-depth analysis of the biological responses of HSCs to mechanical stimulation aims to lay the foundation for developing new and more efficient strategies for erythrocyte production based on biomechanical principles, thereby directly addressing the shortage of donor RBCs.

Bone marrow hematopoietic stem cells (BMHSCs) reside in a specialized three-dimensional (3D) microenvironment (niche) [8,9,10,11], which regulates self-renewal and lineage commitment through biochemical and biophysical cues [12]. Although cytokine-based liquid cultures, such as erythropoietin (EPO), stem cell factor (SCF), interleukin-11 (IL-11), Fms-like tyrosine kinase 3/fetal liver kinase-2 (Flt3/Flk-2) [13,14,15], or extracellular matrix (ECM) protein coatings (collagen, laminin, and fibronectin [12]), partially support erythropoiesis, these systems fail to achieve the scale and speed required for clinical RBCs production due to the following critical limitations: (1) the absence of 3D spatial organization disrupts cell-ECM mechanotransduction essential for terminal erythropoiesis [16,17,18]; (2) homogeneous biochemical environments prevent recapitulation of niche-specific signaling gradients [19]; and (3) shear stress in suspension cultures impairs enucleation efficiency [20]. Consequently, 3D culture systems mimicking the bone marrow niche have emerged to address these constraints [21]. The 3D culture system is considered more conducive to hematopoietic reconstruction due to its higher space utilization and improved material transport rate [22,23]. However, most current 3D platforms—including those using decellularized ECM or synthetic scaffolds—prioritize biochemical cues over mechanical niche properties [24].

Bone marrow niche guides the design of three-dimensional culture systems, the most important of which is the simulated bone marrow ECM network [25,26,27,28], which provides stability to the intercellular spatial structure and anchoring sites for cell adhesion, migration, and cytokine binding [5,12,29]. However, the components that comprise the ECM are unevenly distributed in the bone marrow. For example, type I collagen and osteopontin are only present in the endosteal regions [30,31], and laminin is detected in the vascular region [32]. These uneven distributions may lead to different functions of bone marrow niches, which are caused by differences in their mechanical properties. Studies have also shown that the hard inner bone region (approximately 25–35 Kpa) supports the quiescent state of HSCs, whereas the softer vascular region (approximately 0.3–2 Kpa) promotes the differentiation and maturation of RBCs—a process that does not occur in uniform liquid culture [33,34,35,36].

To address the critical need for tunable mechanical environments in RBC generation, we strategically selected a gelatin methacryloyl (GelMA)-poly (ethylene glycol) diacrylate (PEGDA) hybrid hydrogel system. GelMA provides essential biochemical cues absent in purely synthetic polymers. For example, its RGD motifs facilitate integrin-mediated adhesion [37]—a mechanism crucial for erythroblast survival, maturation, and enucleation [38,39]. Furthermore, GelMA’s photocrosslinkability enables precise spatial patterning of soft (0.5–2 kPa) vascular-like zones [40,41], recapitulating the mechanical landscape where native erythropoiesis occurs. However, GelMA’s rapid degradation and low strength preclude long-term mechanical stability—a requirement for sustained HSC expansion and differentiation [41]. Conversely, although PEGDA allows independent tuning of stiffness across the physiologically relevant range (0.3–35 kPa) without altering GelMA’s bioactivity [42,43,44], it lacks cell-adhesive motifs [45]. Our GelMA/PEGDA (GMP) composite uniquely integrates both functionalities: GelMA delivers erythroid-specific bioadhesion, whereas PEGDA provides long-term mechanical integrity and precise stiffness control. This synergy creates a biomimetic platform capable of maintaining defined mechanical cues throughout the extended erythroid differentiation timeline.

In this study, scaffolds with a gradient of stiffness were designed to investigate the role of substrate mechanics in directing HSC fate towards erythrocyte differentiation. We hypothesize that scaffolds with lower Young’s modulus will significantly enhance HSC commitment to the erythroid lineage and improve the efficiency of terminal differentiation into enucleated RBCs, compared to stiffer substrates. This prediction is based on the established mechanosensitivity of HSCs and the known influence of soft microenvironments on promoting erythroid progenitor expansion. We expect to observe a quantitative increase in the yield of functional, enucleated RBCs from HSCs cultured on the softest scaffolds. By rigorously testing this hypothesis linking scaffold stiffness to erythroid output, this work aims to provide foundational insights for optimizing the mechanical design of future in vitro hematopoietic systems and ultimately contribute to a new perspective for RBC manufacturing in transfusion medicine.

## 2. Results

### 2.1. Preparation and Properties of Scaffolds

**Figure 1:**
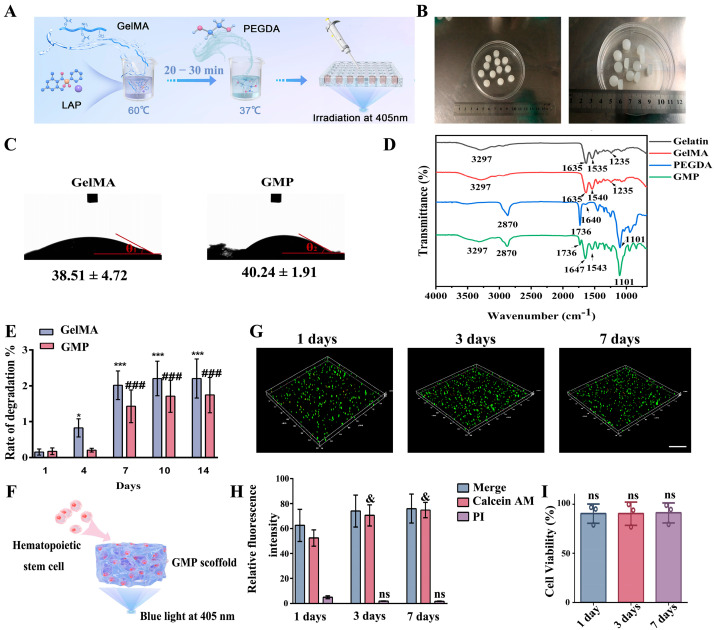
Preparation and performance testing of scaffolding for simulated bone marrow niches. (**A**,**B**) The mechanism of hydrogel formation and the prepared hydrogels. (**C**) Changes in the hydrophilicity of the scaffold after PEGDA was added, *n* = 3. (**D**) Successful preparation of GMP scaffold was measured by infrared spectroscopy. (**E**), Detection of scaffold degradation rate (*n* = 3, in the GelMA group, compared with the first day, * *p* < 0.05, *** *p* < 0.001; In the GMP group, compared with the first day, ### *p* < 0.001). (**F**) Scaffold and cell co-culture diagram. (**G**) Cells cultured in the scaffold were stained alive and dead at days 1, 3, and 7 and imaged under confocal microscopy (scanning height is 100 μm and scanning step size is 8 μm). (**H**) Fluorescence intensity analysis (Compared with the fluorescence intensity of the first day of Calcein AM, & *p* < 0.05; Compared with the fluorescence intensity on the first day of PI, ns means no significant difference, *n* = 3). (**I**) CCK-8 was used to detect cell viability on scaffolds (*n* = 3, there is no significant difference when comparing each pair).

To simulate the internal pore structure of bone marrow in vitro, we used light-curing methacrylic anhydride--modified gelatin as the main material and added short-chain PEGDA to strengthen the cross-linking network. Polymerized crosslinking was performed by light irradiation at 405 nm. The mechanism of hydrogel formation and the prepared hydrogels are shown in Figure 1A,B. The prepared hydrogel has a diameter of 10 mm and a height of 4 mm, as shown in Figure 1B.

As shown in Figure 1D, the gelatin amide A band is 3297 cm^−1^, and the gelatin amide I band is 1635 cm^−1^, primarily associated with the stretching vibration of the C=O bond. The C-H stretching vibration and N-H bending vibration are both manifested at 1535 cm^−1^, representing the amide II band of gelatin. The amide III band, along with CH_2_, N-H, and C-N vibrations appears together at 1235 cm^−1^. The spectral peak shape of GelMA is similar to that of gelatin, but the amide II band is shifted towards high frequency due to the addition of the methylacrylamide group, which is manifested at 1540 cm^−1^. For PEGDA, the main features are the peaks of 1101 cm^−1^, 1736 cm^−1^, and 1640 cm^−1^, where the peak of 1101 cm^−1^ is related to the C-O-C bond, and the peaks of 1736 cm^−1^ and 1640 cm^−1^ are from the C=O and C=C bonds, respectively. Other peaks are also shown in Figure 1, such as the band at 2870 cm^−1^ associated with the vibration of the C-H bond. Finally, GMP, successful expression of gelatin main groups, methyl acrylamide, and the main group of PEGDA, indicates successful material synthesis.

Figure 1C shows that there is no significant difference in the hydrophilicity of the scaffold compared with GelMA after the addition of PEGDA, indicating that GMP is a hydrophilic material.

We chose two weeks as the time point to investigate the degradation rate. The results in Figure 1E shows that after one week, GMP started to experience degradation. However, the final degradation rate was not higher than 5%, which met our experimental needs.

The cells were mixed with the scaffold solution, and light was used to form the composite material with an aperture structure (Figure 1F). Then cells from days 1, 3, and 7 were collected for live/dead assays (Figure 1G). Relative fluorescence intensity analysis showed that calcein AM fluorescence was enhanced on days 3 and 7 compared with day 1 (*p* < 0.05) (Figure 1H), whereas PI fluorescence intensity did not significantly differ. Analysis cell viability and CCK8 assay results showed (Figure 1I) greater than 90% cell viability (90.21 ± 9.86; 90.27 ± 11.54; 91.04 ± 10.22). In addition, the cells were evenly distributed on the scaffold.

### 2.2. Preparation of Scaffolds with Varying Matrix Stiffness

**Figure 2:**
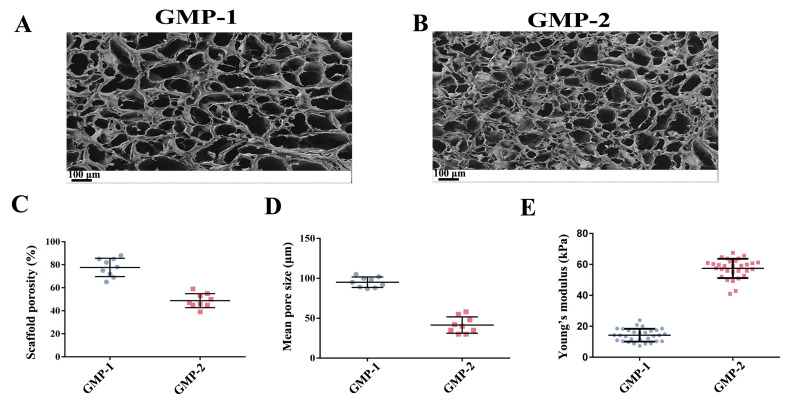
Preparation of scaffolds with different matrix stiffness. (**A**,**B**) SEM images of scaffold with different crosslinking times (scale bar = 100 μm). (**C**,**D**) Mercury intrusion porosimetry measured the porosity and mean pore size of the two scaffolds (*n* = 3). (**E**) AFM detects the Young’s modulus values of two scaffolds (*n* = 30).

The internal structure of the scaffold was assessed using scanning electron microscopy (SEM) (Figure 2A,B). To obtain the average pore size and porosity of GMP scaffolds, mercury injection experiments were conducted (Figure 2C,D). The average pore size of the GMP–1 scaffold was 100 μm with an average porosity of 80%, and the average pore size of GMP–2 was 30 μm. The average porosity was 40%. The Young modulus of GMP–1 and GMP–2 were 14.20 ± 4.32 kPa and 57.68 ± 6.23 kPa, respectively, as measured using atomic force microscopy (Figure 2E). The growth of cells implanted in different scaffolds was photographed using a cryobiomicroscope.

### 2.3. Culture LSKs in Scaffolds with Varying Young’s Modulus

**Figure 3:**
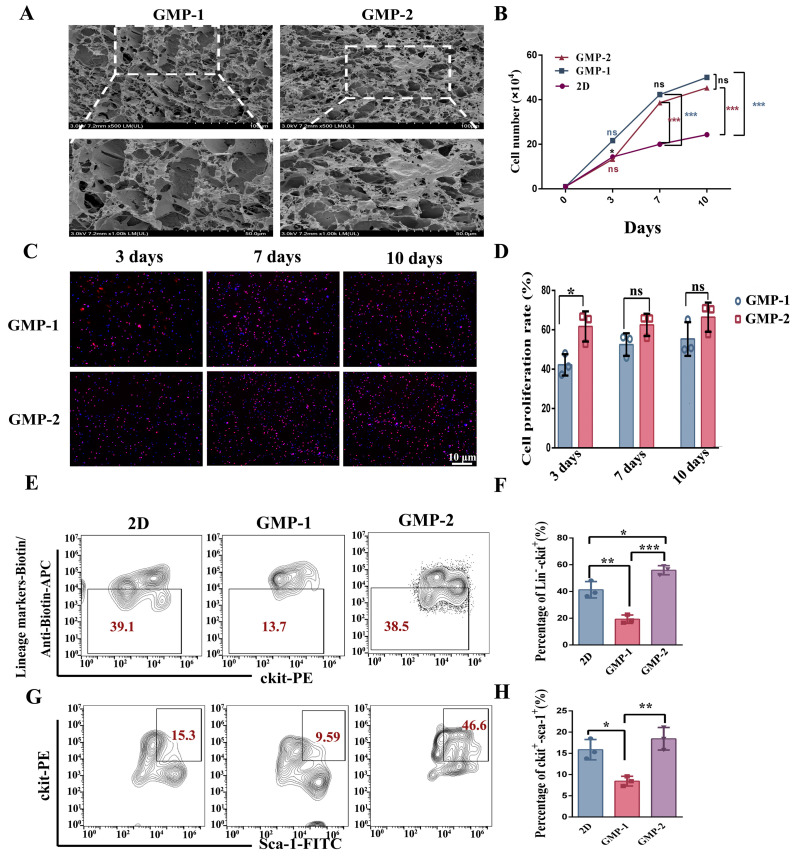
Cell growth on scaffolds with different stiffness. (**A**) Cryo-SEM images of cells cultured on scaffolds for 7 days. (**B**) Cell growth curve (*n* = 3, ns indicates no significant difference). (**C**,**D**) EDU cell proliferation staining analysis (*n* = 3, scale bar = 10 μm, * *p* < 0.05, ns indicates no significant difference.). (**E**,**G**) Flow cytometry analysis of Lineage^−^, ckit^+^, sca-1^+^ cells cultured for 7 days on scaffolds with different Young’s modulus. (**F**,**H**) Expression of Lineage^−^, ckit^+^, sca-1^+^ were analyzed and detected (*n* = 3, * *p* < 0.05, ** *p* < 0.01, *** *p* < 0.001).

Cryo-SEM was used to detect the growth of cells on the scaffold. As shown in Figure 3A, the cells grew well in the scaffolds and were evenly distributed.

An analysis based on cell count results, indicate that when the initial cell number and the days of expansion culture were the same, the cell counts of GMP–1 and GMP–2 after 3 days of culture showed no significant difference compared to the 2D environment (Figure 3B). However, the number of cells obtained after the 7th and 10th days were significantly higher than in the 2D liquid culture environment (*p* < 0.001, *p* < 0.001; *p* < 0.001, *p* < 0.001), and the cell growth volume increased by 2–3 times compared to the 2D culture. The cell counts on the third day showed a significant difference between GMP−1 and GMP−2 (*p* < 0.05).

After the cells and scaffold were co-cultured for 3, 7, and 10 days, EDU cell proliferation detection was performed. Fluorescence staining and cell proliferation rate analysis, revealed that the proliferation rate of LSK cells on the third day was significantly higher on the GMP–2 scaffold compared with the GMP–1 (*p* < 0.05) (Figure 3C,D). No significant difference was noted between the two cultures on the 7th and 10th days.

Cells cultured on scaffolds for 7 days were labeled with stem cell surface markers (lin^−^, ckit^+^, sca-1^+^), and the ratio of stem and progenitor cells obtained in three scaffolds was evaluated using flow cytometry (Figure 3E,G). The results showed that cells obtained from GMP−2 maintained a higher proportion of progenitor cell surface markers than those obtained from GMP−1, while cells obtained from the soft matrix showed a tendency to differentiate (Figure 3F,H) (*** *p* < 0.001, ** *p* < 0.01, * *p* < 0.05).

### 2.4. Matrix Stiffness Promotes Early Erythroid Progenitor Cell Differentiation and Proliferatio

**Figure 4:**
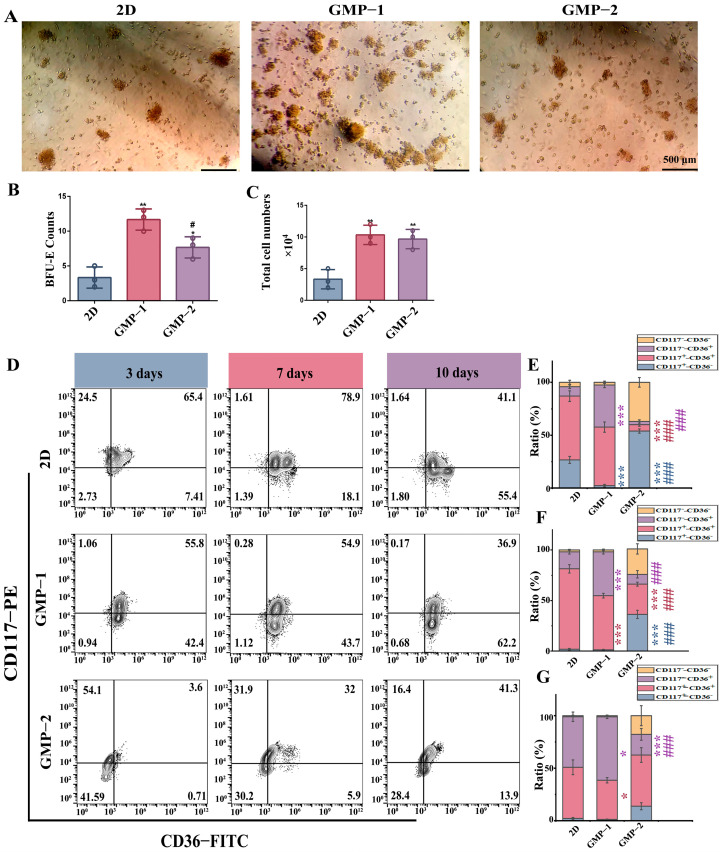
Matrix stiffness promotes differentiation and proliferation of early erythroid progenitor cells. (**A**) Proliferation and quantity statistics of BFU-E (scale bar = 500 μm); (**B**,**C**) BFU-E and total number of cells on the semi-solid medium (compared with 2D, * *p* < 0.05, ** *p* < 0.01; compared with GMP−1, # *p* < 0.05, *n* = 3); (**D**–**G**) The expression of CD117−CD36 surface marker were analyzed and detected by flow cytometry on days 3, 7 and 10 of culture respectively (*, compared with 2D, #, compared with GMP−1, colors represent different quadrants, * *p* < 0.05, *** *p* < 0.001; ### *p* < 0.001, *n* = 3).

The number of BFU-E colonies reflects the proliferation of erythroid progenitor cells, and CD36 expression can be used to characterize the stage of differentiation of erythroid progenitor cells. Figure 4A shows that the size and number of BFU-E colonies formed by cells cultured differed in three environments. Among them, the number of colonies was the largest in GMP−1. Figure 4B shows, the number of colonies formed by cells cultured in scaffolds was significantly higher than that in the liquid environment (** *p* < 0.01, * *p* < 0.05), and GMP−1 was significantly higher than GMP−2 (# *p* < 0.05). The total number of cells on the semi-solid was collected (Figure 4C). It was found that the cells co-cultured with GMP−1 had a greater number of cells, suggesting that GMP−1 promotes early progenitor cell proliferation. Through flow cytometry analysis of CD36 (Figure 4D), CD36^+^ cells cultured on GMP−1 was significantly higher than 2D and GMP−2 on the 3th (Figure 4E), 7th (Figure 4F), and 10th days (Figure 4G). Compared to the liquid environment, the cells cultured in the GMP−1 scaffold differentiate into erythroid progenitor cells more quickly and efficiently.

### 2.5. The Influence of Mechanism Stiffness on the Differentiation Efficiency of Erythroid Cells and Enucleation of Terminal Cells

**Figure 5:**
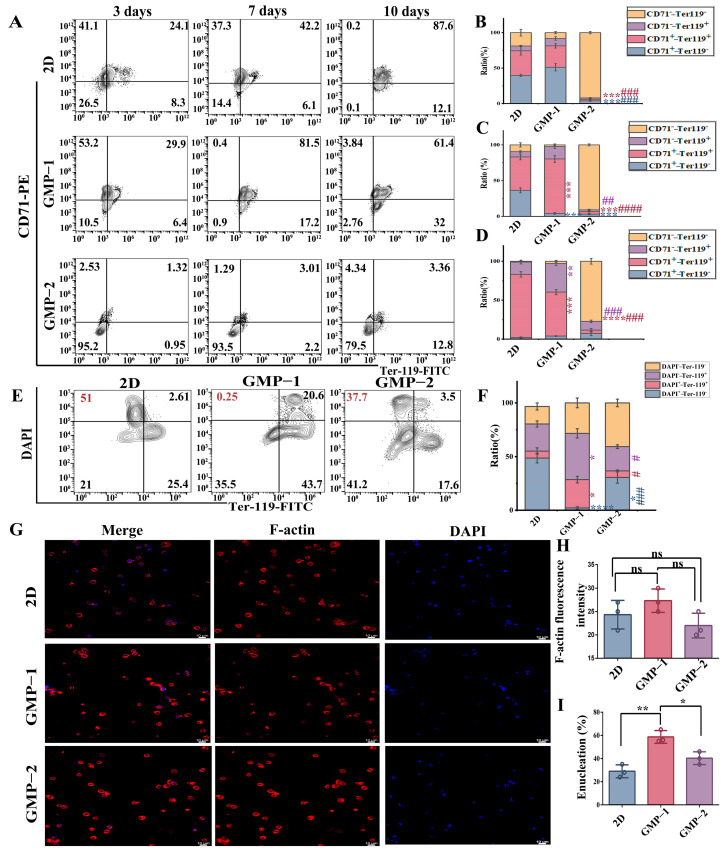
The influence of mechanism hardness on the differentiation rate of erythroid cells and the denucleation of terminal cells. (**A**–**D**) The expression of CD71−Ter−119 surface marker was analyzed and detected by flow cytometry on days 3, 7 and 10 of culture respectively (*, compared with 2D, #, compared with GMP−1, colors represent different quadrants, ** *p* < 0.01, *** *p* < 0.001, **** *p* < 0.0001; # *p* < 0.05, ## *p* < 0.01, ### *p* < 0.001, #### *p* < 0.0001, *n* = 3); (**E**,**F**) Expression and analysis of DAPI−TER−119 in cells on the 14th day of in vitro culture (*, compared with 2D, #, compared with GMP−1, colors represent different quadrants, * *p* < 0.05, **** *p* < 0.0001; # *p* < 0.05, ### *p* < 0.001); (**G**) On the 14th day of in vitro cell culture, the skeletal F-actin protein and the cell nucleus were stained; (**H**) Analysis of the average fluorescence intensity of F-actin protein, ns indicates no significant difference. (**I**) The proportion of cell de-nucleation was calculated based on the staining results (*n* = 3, compared with 2D, ** *p* < 0.01, * *p* < 0.05).

CD71 is continuously expressed throughout the erythroid differentiation process, and ter-119 is highly expressed in the late stage of erythroid differentiation and is the main surface marker of mature erythroid cells. The entire erythroid differentiation process can be tracked through co-staining with CD71−Ter−119. The expression of CD71 and ter−119 was assessed (Figure 5A). CD71^+^−TER−119^+^ cells significantly increased in GMP−1 cultures compared with that in 2D and GMP−2 culture on the 7th day (Figure 5C), and increased CD71^+^−ter-119^+^ and CD71^−^−ter-119^+^ cells were noted in GMP−1 cultures compared with in GMP−2 and 2D culture on the 10th day (Figure 5D). Flow cytometry showed that the proportions of CD71 and Ter−119 expression in GMP−2 cultures was significantly lower than that in GMP−1 and 2D cultures. These results indicate that the cells cultured on GMP−2 were still at the early erythroid progenitor cell stage (Figure 5B–D).

Then, we assessed the terminal differentiation of cells cultured in vitro for 14 days. As shown in Figure 5E, after the cells were stained with Ter−119 and nuclear staining, flow cytometry was used for analysis. The statistical results showed (Figure 5F) that the GMP−1 culture had a higher denucleation rate than the 2D and GMP−2 culture, compared with GMP−2 and 2D, GMP−1 shows a decrease in dapi^+^ cells and an increase in Ter−119^+^ cells (**** *p* < 0.0001, * *p* < 0.05, ### *p* < 0.001; * *p* < 0.05, # *p* < 0.05).

The F-actin protein persists throughout the entire process of erythroid differentiation and does not disappear even after the cells excrete nuclei. Immunofluorescence staining was performed on F-actin and the cell nucleus (Figure 5G, H), and the average fluorescence intensity was analyzed. On the 14th day of differentiation, no significant difference in the average fluorescence intensity of F-actin was observed. Then, statistical analysis of enucleated cells was conducted on the obtained fluorescence images, revealed that GMP−1 cultures had a greater number of enucleated cells than GMP−2 and 2D cultures (Figure 5I, ** *p* < 0.01; * *p* < 0.05).

### 2.6. Matrix Stiffness Improves Erythroid Differentiation After Cytokine Restriction

**Figure 6:**
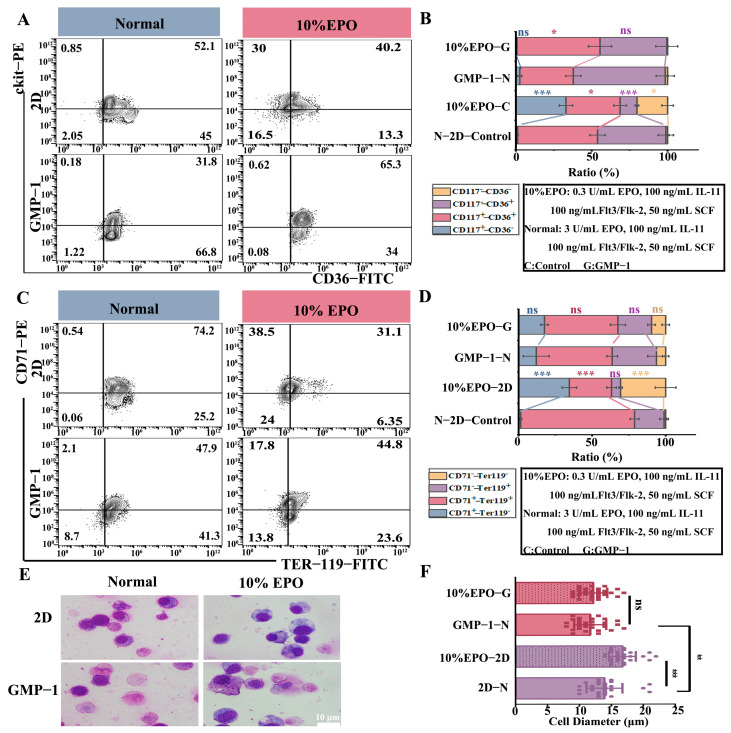
Appropriate matrix stiffness improves erythroid differentiation after cytokine restriction. (**A**,**C**), Flow results of erythroid surface markers ((**A**) ckit-CD36; (**C**) CD71-TER-119) under normal culture and restricted EPO in a liquid environment, and GMP−1 on day 10. (**B**,**D**) Analysis of surface markers (* *p* < 0.05, *** *p* < 0.001, ns indicates no significant difference, *n* = 3). (**E**) Mechanism hardness significantly improved erythroid differentiation in cell morphology—cells under both culture conditions were stained with Wright-Giemsa. (**F**) Calculate the diameters of enucleated cells in each group in Figure 6E (** *p* < 0.01, *** *p* < 0.001, ns indicates no significant difference).

The 2D and GMP−1 cultures were used to detect the expression of CD36, CD71, and ter−119 in under both normal and restricted EPO (10% EPO) culture conditions (Figure 6A,B). Figure 6C shows that 2D culture was strictly dependent on cytokines. When EPO concentration was reduced, CD36^+^ expression decreased significantly, and approximately 40% of the cells were in the stage of hematopoietic stem progenitor cells. After reducing EPO concentration, the differentiation of cells in GMP−1 cultures did not significantly differ from that of normal cultured cells. As shown in Figure 6D, after the reduction of EPO, the differentiation of 2D cultured cells into erythroid progenitor cells was reduced, whereas the differentiation of cells cultured on GMP−1 after the reduction of EPO was not significantly different from that of normal cultured cells on GMP−1.

Further investigation by Wright-Giemsa staining showed that the liquid environment depended on the concentration of cytokines, and scaffold culture could significantly improve the erythroid differentiation process limited by cytokine reduction (Figure 6E,F).

### 2.7. Transplantation Experiment of NOD/SCID Mice In Vivo

**Figure 7:**
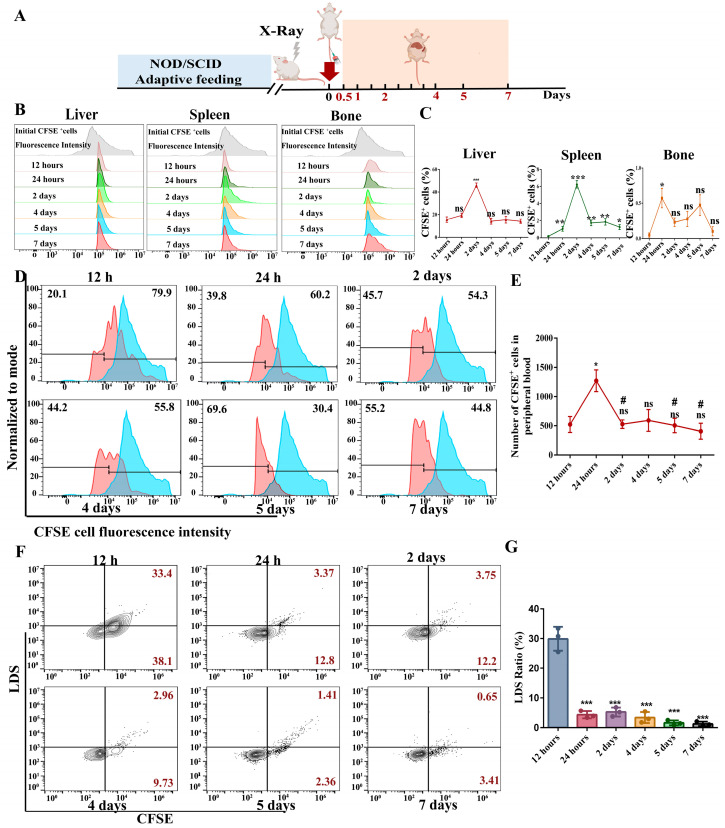
The transplantation experiment evaluated the in vivo fate of the obtained cells. (**A**) An experimental overview diagram. (**B**,**C**) The expression of CFSE^+^ cells in liver, spleen, and bone marrow was detected at different time periods (compared with 12 h, * *p* < 0.05, ** *p* < 0.01, *** *p* < 0.001, *n* = 3). (**D**) Fluorescence intensity of CFSE^+^ cells in peripheral blood at different time periods. (**E**), Proportion and number of CFSE^+^ cells in peripheral blood at different time periods (compared with 12 h, * *p* < 0.05, ns = no significant difference; compared with 24 h, # *p* < 0.05, ns = no significant difference, *n* = 3). (**F**,**G**) The proportion and analysis of cell enucleation in CFSE cells (compared with 12 h, *** *p* < 0.001, *n* = 3).

To track the fate and detect the activity of cells obtained from the in vitro culture system, we injected 5 × 10^5^ CFSE-labeled cells into sublethally irradiated NOD/SCID mice. An experimental overview is shown in Figure 7A. Interestingly, labeled cells were detected one week after injection in all organs examined (liver, spleen, bone marrow, and peripheral blood, Figure 7B–E). As a result, CFSE^+^ cells were not completely cleared, such as the liver or spleen, as expected **(**Figure 7B,C). Similarly, CFSE^+^ cells were detected in the peripheral blood of mice, and the injected cells proliferated in the mice (Figure 7D). In addition, the number of CFSE^+^ cells in the peripheral blood was calculated based onflow cytometry results. The number of CFSE^+^ cells in the peripheral blood increased within 24 h of injection, then decreased, and reached a stable state (Figure 7E). Finally, we detected the nucleation of CFSE cells in peripheral blood. As the time of the cells in the body increased, it was observed that the proportion of LDS^+^ in CFSE cells gradually decreased, suggesting that the injected cells could undergo maturation and denucleation in vivo (Figure 7F). CFSE and LDS expression before injection is reported in the Supplementary Materials (Figure S4).

### 2.8. Cell Function Acquisition

**Figure 8:**
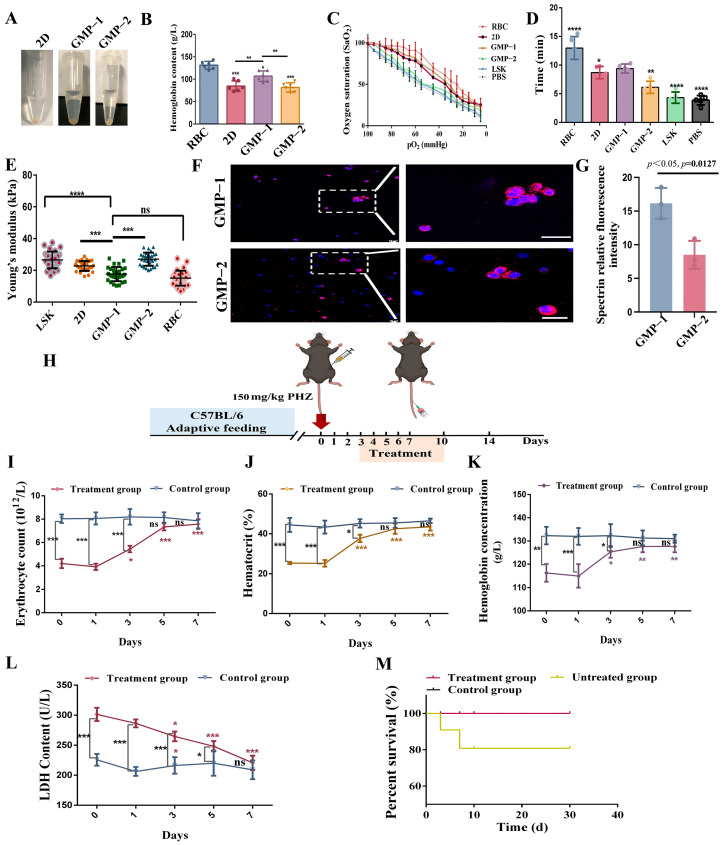
Cell function test. (**A**) Cell precipitation; (**B**) Detection of cell hemoglobin content (compared with RBC, * *p* < 0.01, ** *p* < 0.01, *** *p* < 0.001, *n* = 6); (**C**) Cell oxygen dissociation curve; (**D**) The time it takes for a cell’s oxygen pressure to reach 50% (compared with GMP−1, * *p* < 0.05, ** *p* < 0.01, **** *p* < 0.0001, *n* = 3); (**E**) Acquisition of cell deformability (compared with GMP−1, *** *p* < 0.001, **** *p* < 0.0001, *n* = 3). (**F**,**G**) Protein immunofluorescence staining analysis of spectrin (*n* = 3, * *p* < 0.05). (**H**) Experimental schematic diagram, the function of the detected cells in vivo was restored through the phenylhydrazine hemolysis model; (**I**–**L**) Changes in the number of red blood cells, hematocrit, hemoglobin content and LDH content in the peripheral blood of mice after cell therapy (* *p* < 0.05, ** *p* < 0.01, *** *p* < 0.001, ns indicates no significant difference, *n* = 3). (**M**) Long-term safety assessment of cells.

As shown in Figure 8A,B, cells cultured under different conditions for 14 days were collected and centrifuged to measure hemoglobin content. The hemoglobin content of GMP−1 cultured cells was significantly increased compared with that of 2D and GMP−2 cultured cells (*p* < 0.01, *p* < 0.01). Then we detected the oxygen-carrying and releasing capabilities of cells in different groups. Figure 8C shows that the oxygen dissociation curves of GMP−1 cultured cells were similar to normal red blood cells. The rate of oxygen release is related to the conformational changes of hemoglobin. We assessed the time required for cells in different groups to reach 50% oxygen pressure. As shown in Figure 8D, GMP−1 cells had a slower oxygen release rate than those cultured on GMP−2 and LSK cells (*p* < 0.01, *p* < 0.0001).

The acquisition of erythrocyte deformability is also an important milestone in the differentiation process. The acquisition of cell deformability is the result of the continuous assembly of membrane proteins onto the cell membrane during the differentiation process. As shown in Figure 8E, on day 14 of culture, the Young’s modulus of GMP−1 was significantly lower than that of LSK cells (*p* < 0.001). In addition, the Young’s modulus of GMP−1 was significantly lower than GMP−2 (*p* < 0.001). The decrease of Young’s modulus indicates that the differentiation leads to the nucleus moving to the cell edge or to denucleation. Then, erythrocyte membrane proteins were stained (spectrin, Figure 8F). The results showed that the skeletal proteins continuously assembled onto the membrane during erythroid differentiation, and the cells on GMP−1 exhibited a stronger spectrin fluorescence intensity (Figure 8G).

Finally, we used the phenylhydrazine-hemolytic mouse model and injected differentiated late erythrocyte cells for infusion treatment (Figure 8H). After the treatment, the number of red blood cells, hematocrit and hemoglobin content in the peripheral blood of the mice were significantly increased compared with the hemolysis state and returned to the normal state (Figure 8I–K); Hemolysis leads to an increase in LDH content, suggesting an increase in anaerobic glycolysis levels in mice. After cell infusion (Figure 8L), LDH levels returns to normal levels, indicating that the infused cells can alleviate the hypoxic state of mice. This finding indicates that cells cultured in vitro can play the role of “oxygen carriers” in vivo. After the cell transplantation experiment was completed, the mice were observed for one month (Figure 8M). During the observation period, the mice’s diet and body weight were normal. The regular physical examination results were stable, and no visible lesions were observed. The parameters used for the establishment of the phenylhydrazine hemolytic model can be found in the Supplementary Materials (Figure S5).

## 3. Discussion

This study demonstrates that matrix stiffness is a key physical regulator of erythroid differentiation in HSCs, using GelMA-PEGDA hydrogel scaffolds with distinct stiffness profiles. Both stiff (GMP−2) and soft (GMP−1) scaffolds significantly enhanced cell proliferation compared to conventional 2D culture. However, the scaffolds exerted divergent effects on cell fate: the stiff matrix GMP−2 preserved stemness, whereas the soft matrix GMP−1 efficiently promoted erythropoiesis. Importantly, we established a clear inverse relationship between matrix stiffness and enucleation efficiency. Critically, the pro-differentiation effect of the soft matrix was maintained under erythropoietin (EPO)-limited conditions, indicating its reduced dependence on high cytokine concentrations. This finding suggests a potential strategy for overcoming clinical EPO resistance. Finally, erythroid cells differentiated in vitro within the soft matrix were observed continued proliferation, differentiation, and enucleation capabilities in vivo following transfusion, coupled with potential physiological recovery. This represents a novel approach for generating functional red blood cells.

The successful synthesis of GMP is the foundation for the construction of this bone marrow engineering platform. GMP retains the characteristic groups of gelatin, ensuring the biological activity of cell recognition site. In addition, GMP has the controllability and adjustable range of stiffness of photo-crosslinking, while retaining biological signals and enhancing mechanical stability. Mechanical environment in bone marrow is highly heterogeneous (central bone marrow is 1–10 kPa, near the cortical bone is 10–100 kPa) [46,47,48], and we have simulated this phenomenon. Research has found that the stiff GMP−2 matrix effectively preserves stem cell quiescence by maintaining high LSK cells phenotype, mimicking the stem cell maintenance function of high-stiffness perivascular niches in bone marrow. Prior studies have shown that CXCL12 is located in the perivascular sinusoid wall niches and maintains the quiescent state of HSC [49,50]. In addition, an increase in matrix stiffness inhibits the expression of CXCL12, causing HSCs to enter the proliferative state [51]; Conversely, the soft GMP−1 matrix efficiently simulates the low-stiffness microenvironment beside the blood sinuses, promoting the differentiation and maturation of the erythroid system. This is consistent with the previously published conclusion [6,52,53]. The denucleation rate we reported is higher than that of the liquid culture, and even higher than that of the liquid culture after cytokines optimization [54].

The differentiation advantage of soft matrices was further evidenced by their tolerance to growth factor fluctuations. Under EPO-limited conditions, compared with 2D culture, GMP−1 improved erythroid differentiation ability, indicating that mechanical signaling can compensate for biochemical signal dependency in conventional systems. This characteristic overcomes the limitation of the traditional system in requiring high concentrations of EPO [55], and holds translational potential for treating EPO-resistant anemia [56]. Following in vivo transfusion revealed that these differentiated cells were not immediately ‘cleared’. Rather, these cells remained detectable in mouse tissues and peripheral blood. Previous studies also indicated that macrophages in NOD/SCID mice do not rapidly eliminate injected syngeneic cells [54,57]. The percentage of CFSE^+^ cells peaked within 24 h, suggesting that nucleated cells can continue to rapidly expand in vivo. Enucleated cells entered the bloodstream as detected via splenic examination, and the circulating CFSE^+^ cells (CFSE^+^/LDS^–^) likely represent enucleated red blood cells generated in vivo from the infused immature erythroid cells. These cells facilitate potential physiological recovery in an acute phenylhydrazine-induced hemolysis model, yielding an improvement trend in survival status was observed.

It is necessary to admit that the sample size of the in vivo experiments in this study (*n* = 3) has inherent limitations in terms of statistical power. Although the data show a consistent trend, the small sample size may lead to bias in the assessment of effect size. Therefore, the cell function after in vitro transplantation should be regarded as a preliminary finding and requires validation with a larger sample size.

Overall, this study established an innovative platform for efficiently directing the erythroid differentiation of hematopoietic stem cells by engineering key physical properties of the bone marrow microenvironment. Its core contribution lies in establishing the pivotal role of matrix stiffness in erythroid differentiation. By leveraging this principle, we achieved a breakthrough in erythrocyte production efficiency, specifically overcoming the dependence on high EPO concentrations and addressing the critical bottleneck of low enucleation efficiency. This work provides a solid technical foundation for the scalable, high-quality production of red blood cells in vitro. Furthermore, it deepens our understanding of how physical cues in the bone marrow microenvironment govern hematopoietic stem cell fate decisions and terminal erythrocyte maturation, successfully demonstrating the utility of an engineered hydrogel-based biomimetic platform.

## 4. Conclusions

This study definitively establishes extracellular matrix stiffness as a crucial physical regulator determining HSC fate. We have successfully developed a stiffness-partitioned, biomimetic culture system: stiffer substrates effectively recapitulate the rigid bone marrow microenvironment, preserving HSC stemness, while softer substrates significantly enhance the efficiency and maturity of erythroid differentiation, sustaining effective erythropoiesis even under restrictive erythropoietin conditions. This reveals the unique potential of mechanical signals to compensate for insufficient key biochemical cues. Critically, the erythroid cells induced on the soft substrate exhibit potential physiological functional properties. This stiffness-based regulatory strategy not only provides significant insights into the fundamental mechanisms by which the physical microenvironment governs HSC fate decisions but also demonstrates compelling dual application potential: stiffer matrices for HSC expansion and softer matrices for opening an attractive new pathway towards clinical-scale, scalable red blood cell production.

## 5. Materials and Methods

### 5.1. Isolation and Culture of Hematopoietic Stem Cells 

Lineage^−^ Sca-1^+^ c-Kit^+^ cells (LSK cells, purity = 85.2% ± 9.25) were obtained by immunomagnetic bead sorting. They were cultured in SFEM medium (Stem Span™ Serum-Free Expansion Medium, SFEM, Stem Cell, Vancouver, BC, Canada), with 100 ng/mL IL-11, 50 ng/mL SCF, 100 ng/mL Flt3/Flk-2, 3 U/mL EPO, 40 ng/mL low-density lipoproteins (all from Stem Cell, Vancouver, BC, Canada) and penicillin/streptomycin [58,59,60]. Cells were cultured at 37 °C and 5% CO_2_, and half of the medium was replaced every three days. IL-11, SCF, Flt3/Flk-2, and low-density lipoproteins were added for cell proliferation, whereas EPO was used for differentiation culture. (further information about the LSK is available in Figure S1 of the Supplementary Materials).

### 5.2. Preparation of Marrow-Mimetic Hydrogel Platforms—GelMA-PEGDA

GelMA (Mw = 91.74 kDa, DS = 77%) was prepared as described previously [61]. Briefly 0.6 g GelMA was dissolved in 10 mL lithium phenyl-2,4,6-trimethylbenzoyl hypophosphonic acid (GelMA, 6% *w*/*v*, LAP, 0.25% *w*/*v*) at 50 °C for 20–30 min, with the mixture being shaken several times until completely dissolved. Then, 0.15 g PEGDA (1.5% *w*/*v*) was added, and the mixture was stirred thoroughly. The prepared solution was always maintained at 37 °C in an ultra-clean bench filtered through a 0.22 μm sterile filter membrane. and placed into a mold. It was then irradiated with a 405 nm light source to form a gel. The degree of crosslinking of photocrosslinked materials is related to the illumination time [62]. Based on this, we obtained scaffolds with different crosslinking degrees GMP−1 (5 s) and GMP−2 (20 s) by changing the illumination time

### 5.3. 3D Cell Culture in Scaffolds

The prepared LSK cell suspensions were added to the surface of sterile, dry porous scaffolds, and the cells were seeded and incubated at 37 °C and 5% CO_2_ for 30 min, allowing the cell suspensions to be completely absorbed into the scaffold pores LSK cells were maintained at 1 × 10^5^ cells/scaffold [63,64,65]. Subsequently, the medium without cytokines was added and cells were incubated for 30 min. Finally, it was replaced with the SFEM medium without EPO. After 2 to 3 days of adaptation culture, 3 U/mL EPO or 0.3 U/mL EPO (restricted EPO experiment) was added to the initial medium, and the medium was changed every 2 days.

To release the cells, the gel was cut into small pieces, washed with PBS, and incubated with trypsin/EDTA (Gibco, Grand Island, NY, USA) while shaking. The cells were collected by centrifugation.

### 5.4. Fourier-Transform Infrared (ATR-FTIR)

The prepared gelatin, GelMA, and GMP scaffolds were frozen at −80 °C overnight and then freeze-dried in a freeze dryer for 48 h (−50 °C, 0.1 mpa). The infrared spectrum of freeze-dried samples was measured by ATR-FTIR (Nicolet iN10, Waltham, MA, USA) mode between 4000 and 700 cm^−1^.

### 5.5. Material Hydrophilicity Test

Dataphysics (Filderstadt, Baden-Württemberg, Germany, OCA20) was used to measure the change in hydrophilicity of the scaffolds after adding PEGDA. Firstly, GelMA and GMP scaffolds with smooth and flat surfaces were prepared (1 × 1 × 0.4 cm). Samples were placed on the contact angle test platform, and droplets were put on the device’s automatic titration system. Test photos were taken, and the contact angle was measured [66,67].

### 5.6. Detection of Scaffold Degradation Rate

The prepared scaffolds were placed in the culture medium for 24 h, after which they were removed and weighed (W_0_). Then scaffolds with different culture days were weighed (W_1, 2 … n_). The degradation rate of scaffold can be calculated according to the formula [68]:

$$W\%=\frac{W_{0}-W_{1,2\ldots n}}{W_{0}}\times 100\%$$

### 5.7. Young’s Modulus

To measure scaffold elasticity, it was freeze-dried and then examined using atomic force microscopy (AFM, BRUKER, Billerica, MA, USA).

After a cell suspension was generated, the cells with adhesive properties were added to a glass slide.

When measuring the scaffolds and cells, we used a soft-cone silicon nitrided cantilever with elastic constants of 0.4 N/m (F) and 0.92 N/m (F), a probe angle of 18°, and a probe radius of 23.1 nm for the measurements. We determined the Young’s modulus of the scaffold fragments in the DMT modulus channel using Nanoscope analysis software (V3R1sr6).

For each experiment, 30 cells were randomly selected, and 30 points were tested on each scaffold. The observations were conducted under a microscope.

AFM was conducted using the Sneddon model [69] formula as follows:

$$F=\frac{\pi }{2}(\frac{E}{1-v^{2}})tan(\alpha )\delta^{2}$$

where *E* is the elastic or Young’s modulus, *v* is the Poisson ratio (assumed to be 0.5), α is the opening angle, and δ is the indentation depth (100 nm).

### 5.8. Scanning Electron Microscopy (SEM)

SEM (ZEISS Gemini 300, Oberkochen, Baden-Württemberg, Germany) was used to observe the internal structure of scaffolds. Freeze-dried samples (−80 °C) were sputtered using a sputter coater to sputter a layer of gold as a conductive thin layer material for 45 s. Then, SEM was used to scan and image the samples.

### 5.9. Mercury Intrusion Porosimetry (MIP)

The mercury porosimeter, based on the intrusion of mercury into the pores in the materials by applying controlled pressures, was used to analyze the pore structure by tracing the amount of mercury infiltrated at each gas pressure.

### 5.10. Cytocompatibility

Cell viability was evaluated by live/dead cell staining. Calcein AM (Meilunbio, Dalian, China) was used to label viable cells in green, whereas propidium iodide (PI) labeled dead cells. Specifically, scaffolds co-cultured with cells were rinsed with PBS. Then, a staining solution (2 μM calcein AM, 8 μM PI) was prepared and incubated with the scaffolds for 30 min at 37 °C. The scaffolds were washed three times with PBS for2 min each wash at 800 g, and then observed under a laser confocal microscope.

Cell viability was further detected by cell counting kit-8 (CCK-8) assay. Specifically, cells on day 3 of culture were harvested, washed with PBS three times (2 min each, 800 g), and then added to fresh medium containing 10% CCK-8 solution. The cells were incubated for 2 h at 37 °C in a 5% CO_2_ incubator, and the absorbance was measured at 450 nm.

### 5.11. Biocryo-Scanning Electron Microscopy (Cryo-SEM)

The 3D co-culture scaffolds were washed in PBS and then fixed with 2.5% glutaraldehyde for 2 h. The fixed sample was placed on the sample table coated with conductive carbon glue. Then, the sample stage with the fixed sample was placed into liquid nitrogen for 30 s, then subjected to sublimation gold plating (10 Ma, 60 s) under low temperature (−90 °C) and vacuum conditions. Finally, the observation and imaging were carried out. The temperature of the cold stage was −140 °C, and the acceleration voltage was 5 kV.

### 5.12. EDU Detection of Cell Proliferation

Collected the cells from the scaffold, washedthree times with PBS for 2 min each time, then label them with the EDU staining solution. After the labeling completed, washed the cells, and treated with paraformaldehyde (40 min) and Triton X-100 (5 min). Finally, cells were stained with the DAPI staining solution for 10 min. After the staining was completed, use a laser confocal microscope for detection.

### 5.13. BFU-E Clone Formation Experiment

After culturing in vitro for 3 days, 10,000 cells were directly collected, washed with PBS, re-suspended, and mixed in methylcellulose medium. Three replicates of the mixture were placed in 35 mm culture dishes and cultured under standard cell culture conditions for 14 days. The colonies were observed under an optical microscope, and the cells in the culture medium were collected for total cell counting.

### 5.14. Immunofluorescence Staining

Scaffolds for different culture days were removed and washed with PBS. The cells were fixed with 4% paraformaldehyde for 2 h, washed with PBS, permeabilized with 0.5% TritonX-100 for 10 min, washed with PBS, and blocked with 5% bovine serum albumin for 1 h. Next, the primary antibody was used for immunostaining overnight (rabbit monoclonal spectrin antibody, abcam, 1:1000). Then, samples were exposed to fluorescent secondary antibodies at 37 °C for 2 h (Alex 594 goat anti-rabbit, Zenbio,1:200, Chengdu, China). Finally, the nuclei were stained with DAPI (Solarbio, Beijing, China) for 10 min. The samples were placed under a laser confocal microscope (Leica, Wetzlar, Hessen, Germany) for imaging, an imaging objective is 40/0.75.

Collected and washed the cells, fixed the cells on ice with 3.75% formaldehyde for 15 min; Then treat the cells with 0.5% TritonX-100 for 10 min; Then, 200 μL of cell suspension was added to 1 μL of phalloidin solution for 20 min; After PBS cleaning, DAPI was added for final staining., and the samples were observed under a laser confocal microscope.

### 5.15. Flow Cytometry

2D cultured cells were collected, washed with PBS, and added with antibodies (Anti-Lineage-Biotin, Miltenyi Biotechnology, Bergisch Gladbach, Germany; FITC-conjugated Sca-1 antibody, Miltenyi Biotechnology; PE-conjugated c-kit antibody, Santa Cruz Biotechnology, Dallas, TX, USA; FITC-conjugated CD36; PE-conjugated CD71 antibody, Santa Cruz Biotechnology; FITC-conjugated TER-119 antibody, Thermo Fisher Scientific, Waltham, MA, USA; DAPI, Beijing, China), 37 °C incubated for 30 min. For 3D-cultured cells, the scaffold was trimmed into small pieces, digested in trypsin with concomitant gentle blowing, and the cells were collected, after which the procedure was performed as for 2D cells. Stained cells were placed on a flow cytometer (Beckman Coulter, Brea, CA, USA) for analysis, and 10^5^ cells were collected per tube (The gating strategy is presented in the Supplementary Materials, Figure S3).

### 5.16. Hemoglobin Detection

Cells were collected by centrifugation and then washed three times with pre-cooled PBS (2 min/each time). The washed cells were re-suspended in 500 μL of PBS with protease inhibitors and lysed using an ultrasonic cell disruptor (40% amplitude, 3 s/time, intermission time 10 s,10 cycles). All steps were carried out while ensuring that the sample was maintained at 4 °C. The extract was centrifuged at 1500 g for 10 min at 4 °C, and the supernatant was collected for testing.

This experiment adopts the “sandwich-ELISA” principle. Specifically, the sample was added to the wells of the ELISA plate to allow it to combine with the specific antibody. Then, anti-mouse HB biotinylated antibody and HRP enzyme solution were added to each well, and incubation was performed followed by washing. Next, the TMB solution was added to each well. Finally, the reaction was terminated by adding the stop solution, and the optical density (OD) was measured at a wavelength of 450 nm using spectrophotometry.

### 5.17. Oxygen Dissociation Curve

Different groups of cells were washed and adjusted to 0.4 L/L of cell volume, and then 20 μL of fetal bovine serum was added. Put the sample into the sample pool and set the experimental temperature to 37°; For the initial state setting, when mixed air was injected and the partial oxygen pressure of the sample was the same as that of the incoming gas (150 mmHg), the hemoglobin is considered to be completely bound with oxygen, and the oxygen saturation was 100%; Then the oxygen dissociation stage was carried out. Nitrogen was injected into the sample at a constant rate, the changes of oxygen partial pressure (pO_2_) and oxygen saturation (SatO_2_) of the sample were recorded over time, and the oxygen dissociation curve was drawn.

### 5.18. Wright-Giemsa Stain

The cell suspension was collected, evenly coated on the slide, and fixed with methanol. The fixed smear was added with Giemsa dye solution. After dyeing, the dye solution was rinsed, and the smear was dried and observed under a microscope.

### 5.19. Phenylhydrazine Hemolysis Recovery

An acute phenylhydrazine hemolysis model was established. During hemolysis, cells cultured in vitro for up to 14 days were injected intravenously for recovery. The peripheral blood indicators of mice were observed after the injection.

### 5.20. Fate of Erythroid Cells in NOD/SCID Mice

NOD-LtSz-scid/scid (NOD-SCID) mice (7–8 weeks old) were raised under sterile conditions. Before cell injection, X-ray irradiation (2.5 Gy, 2 Gy/min [2]) was performed. The cells were expanded in vitro for 7 days, washed, labeled with CFSE, re-suspended in PBS containing 0.1% BSA, and then injected into the tail vein of mice (5 × 10^5^ cells/each mouse [69]). Three animals were euthanized at each time point, and the spleen, liver, and long bone of the mouse hind limb were taken to make a single-cell suspension, and heparin-anticoagulant venous blood was collected. Fluorescence-activated flow cytometry (FACS) was used to detect the enrichment degree of labeled CFSE^+^ cells and LDS expression in CFSE^+^ cells.

Using unstained cells as negative controls, gating parameters were established. The same experiment was conducted on mice without injected cells to rule out tissue autofluorescence. Before injection, the cells were stained with CFSE and the initial fluorescence intensity was determined by flow cytometry.

### 5.21. Statistical Analysis

All data were expressed as mean ± standard deviation (mean ± SD). Unpaired t-test was used for comparisons between two groups, and one-way or two-way analysis of variance were used for comparison of more than three groups. GraphPad Prism 6.0 software was used for analysis of variance and graph generation. In our study, “n = 3” represents independent biological replicates and the entire research process followed a randomized strategy of grouping, testing, and data analysis.

## Data Availability

The original contributions presented in this study are included in the article/Supplementary Material. Further inquiries can be directed to the corresponding authors.

## Supplementary Materials

The following supporting information can be downloaded at: https://www.mdpi.com/article/10.3390/gels11080594/s1, Figure S1: LSK cell purity detection and gate strategy; Figure S2: Nuclear magnetic hydrogen spectrum of Gelatin and GelMA; Figure S3: Flow cytometry gating strategy; Figure S4: CFSE-LDS Flow Cytometry Gate Strategy; Figure S5: Establishment of acute hemolysis model.

## Abbreviations

The following abbreviations are used in this manuscript:

RBC	red blood cells
HSCs	hematopoietic stem cells
EPO	erythropoietin
SCF	stem cell factor
Il-11	interleukin-11
Flt3/Flk-2	Fms-like tyrosine kinase 3/fetal liver kinase-2
ECM	extracellular matrix
GelMA	gelatin methylallylamine
PEGDA	poly (ethylene glycol) diacrylate
