# Supplementary material for: Engineering Marrow-Mimetic Hydrogel Platforms Enhance Erythropoiesis: A Mechanobiology-Driven Approach for Transfusion Red Blood Cell Production
Source: Gels. 2025 Jul 31;11(8):594. doi: 10.3390/gels11080594 (PMC12385703; doi:10.3390/gels11080594)
Supplement: Supplementary file 1 [file gels-11-00594-s001.zip › gels-3732726-supplementary.pdf]

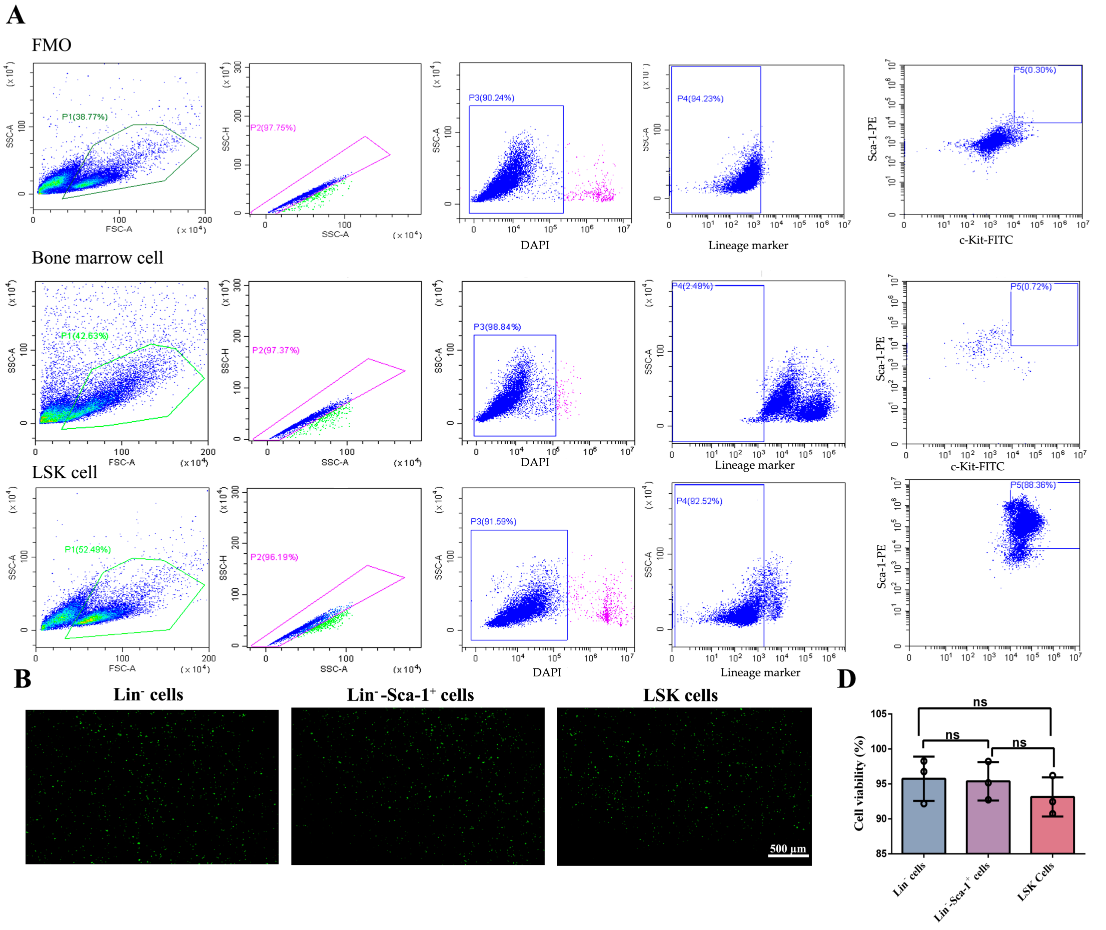

Figure S1. LSK cell purity detection and gate strategy (**A**) The purity percentage of LSK cells obtained through sorting (the percentage of p5 gate cells in the total p3 gate cells). (**B**) Live and dead cell staining for detecting and sorting cell activity. (**C**) Cell viability staining using Calcein AM-PI. (**D**) CCK8 assay for cell viability (*n*=3, ns indicates no significant difference).

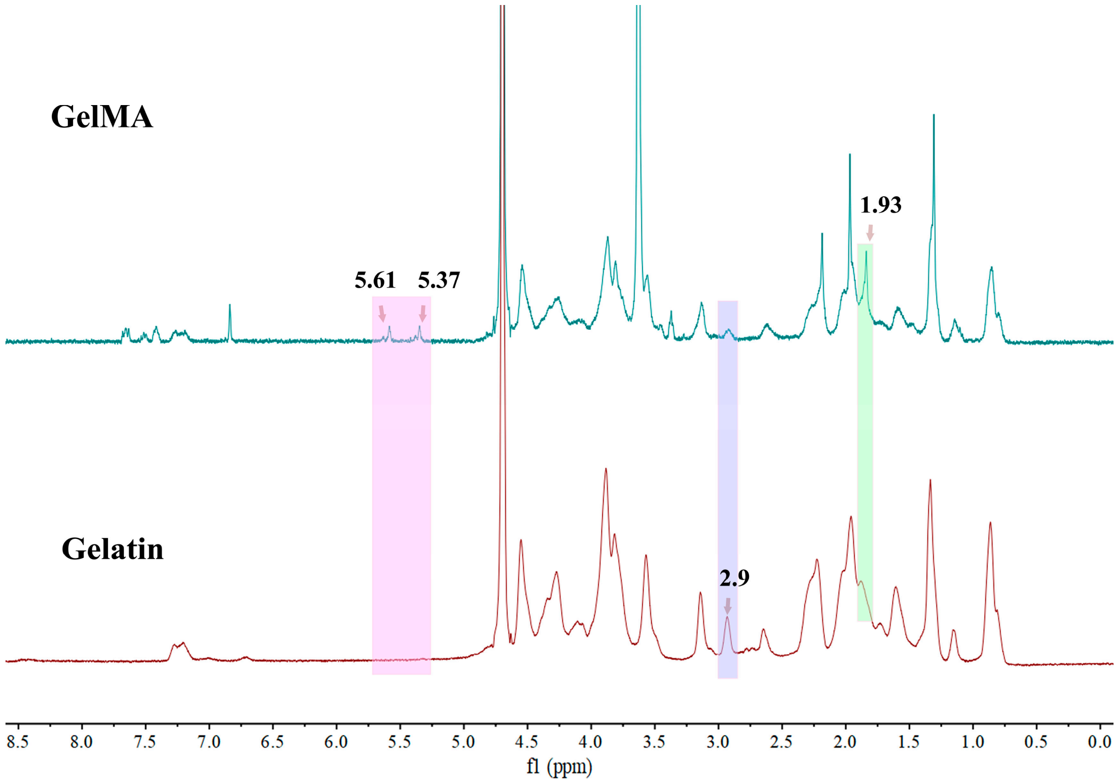

Figure S2. Nuclear magnetic hydrogen spectrum of Gelatin and GelMA

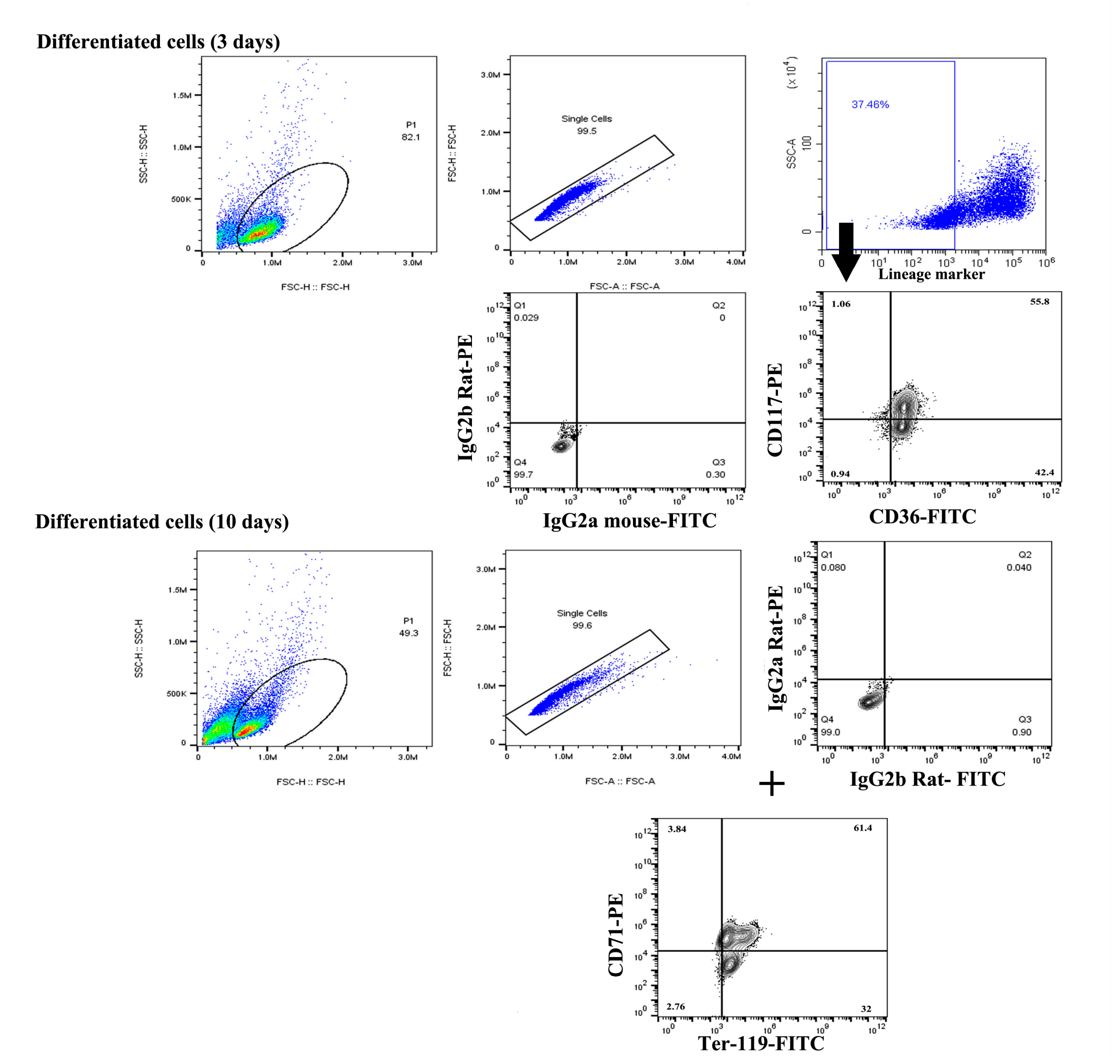

Figure S3. Flow cytometry gating strategy. In the early stage of differentiation, surface marker staining is performed within the Lin⁻ group, and a threshold is set in combination with isotype control. At the advanced stage of differentiation, set the gate with isotype control.

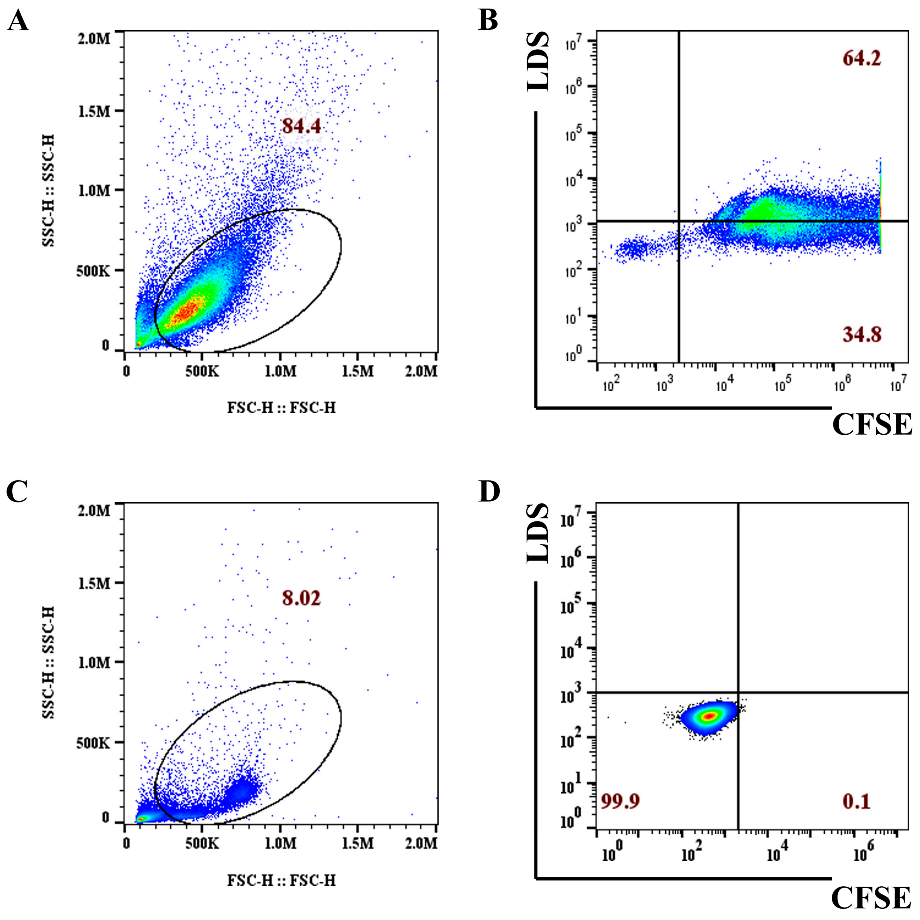

Figure S4. (**A**)Uninjected CFSE cells. (**B**) The expression of LDS in uninjected CFSE cells. (**C**) The FSC-SSC results of peripheral blood from mice with uninjected CFSE cells. (**D**)The results of FSC-SSC in the peripheral blood of mice with uninjected CFSE cells, and the expression of LDS in blank peripheral blood after setting the gate with the results in B.

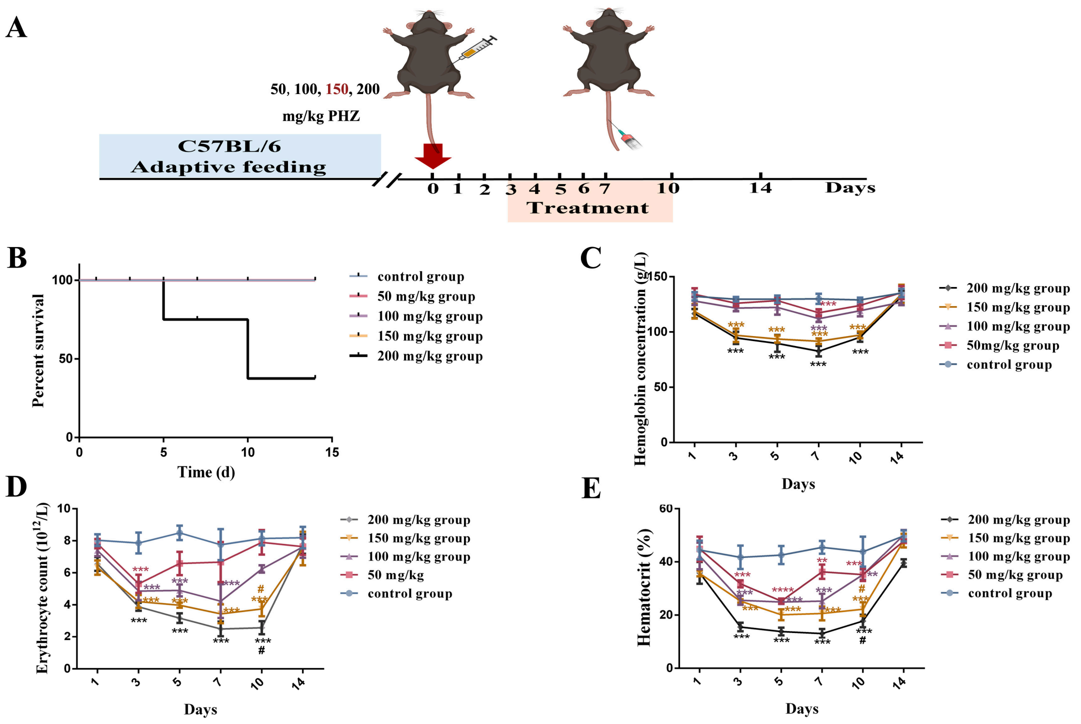

Figure S5. (**A**)Schematic diagram of experimental operation. (**B**)Survival curves of mice after injection of different concentrations of phenylhydrazine. (**C, D, E**) Detection of hemoglobin content, red blood cell count and hematocrit in mice.
